# Chronic Unilateral Proptosis in Primary Care: A Case of IgG4-Related Orbital Disease

**DOI:** 10.7759/cureus.111046

**Published:** 2026-06-17

**Authors:** Yaqeen M Sahwan, Najlaa M Alsudairy

**Affiliations:** 1 General Practice, Dammam Medical Complex, Dammam, SAU; 2 Family Medicine, National Guard Health Affairs, Jeddah, SAU

**Keywords:** computed tomography, diffusion restriction, igg4-related orbital disease, lacrimal gland enlargement, magnetic resonance imaging, orbital lymphoma, orbital mass, orbital pseudotumor, proptosis

## Abstract

Immunoglobulin G4-related orbital disease (IgG4-ROD) is an uncommon fibroinflammatory condition that may closely resemble malignant orbital lesions, posing a significant diagnostic challenge. We report the case of a 50-year-old man who presented with a four-year history of progressive right-sided proptosis. Computed tomography and magnetic resonance imaging revealed enhancing extraconal orbital masses involving the lacrimal gland and extraocular muscles, with imaging characteristics including restricted diffusion that strongly suggested orbital lymphoma. Given the indeterminate radiological findings, the patient underwent anterior orbitotomy and incisional biopsy. Histopathological examination demonstrated dense lymphoplasmacytic infiltration and collagenous fibrosis with abundant IgG4-positive plasma cells and an elevated IgG4/IgG ratio, establishing the diagnosis of IgG4-ROD and excluding malignancy. The postoperative course was uneventful, and further evaluation was arranged to assess for systemic involvement. This case highlights the substantial clinical and radiological overlap between IgG4-ROD and orbital lymphoma and underscores the limitations of imaging in achieving a definitive diagnosis. Recognition of characteristic patterns of orbital involvement, particularly when the lacrimal gland and extraocular muscles are affected, may raise suspicion for IgG4-ROD; however, histopathological confirmation remains essential. Early and accurate diagnosis is critical to avoid misclassification as a malignant process, prevent inappropriate treatment, and ensure appropriate management and surveillance. This report emphasizes the importance of maintaining a broad differential diagnosis when evaluating chronic orbital masses and reinforces the pivotal role of tissue diagnosis in guiding clinical decision-making.

## Introduction

Immunoglobulin G4-related disease (IgG4-RD) is a chronic fibroinflammatory immune-mediated condition characterized by tumefactive lesions, dense lymphoplasmacytic infiltrates rich in IgG4-positive plasma cells, and varying degrees of fibrosis [1-3]. Since its recognition as a distinct clinical entity, IgG4-RD has been reported to involve multiple organs, including the pancreas, salivary glands, lacrimal glands, kidneys, lungs, and lymph nodes. Orbital involvement, referred to as IgG4-related orbital disease (IgG4-ROD), represents a relatively uncommon manifestation and most frequently affects the lacrimal glands, extraocular muscles, trigeminal nerve branches, and orbital soft tissues [3-6]. Patients typically present with slowly progressive proptosis, eyelid swelling, diplopia, or orbital masses, often resulting in significant diagnostic challenges.

The radiologic appearance of IgG4-ROD is highly variable and may closely resemble malignant or inflammatory orbital disorders, particularly orbital lymphoma and idiopathic orbital inflammatory disease [1-4]. Imaging findings often demonstrate enhancing soft-tissue masses with infiltration of adjacent orbital structures, making definitive diagnosis based solely on clinical and radiological features difficult. Histopathological examination remains the diagnostic cornerstone, demonstrating characteristic lymphoplasmacytic infiltration and increased numbers of IgG4-positive plasma cells [3-6]. Early and accurate diagnosis is essential because IgG4-ROD generally responds well to immunosuppressive therapy and has a markedly different prognosis and management strategy compared with orbital malignancies [7-10].

From a family medicine perspective, patients with IgG4-RD may initially present with subtle, slowly progressive symptoms involving a single organ system. Because manifestations are often nonspecific and may evolve over several years, primary care physicians play a critical role in identifying concerning clinical features, initiating appropriate investigations, coordinating specialist referrals, and monitoring for multisystem involvement. Awareness of uncommon conditions such as IgG4-RD is important when evaluating persistent masses, unexplained glandular enlargement, or chronic inflammatory presentations that do not fit common diagnostic patterns. We report a case of biopsy-proven IgG4-ROD that clinically and radiologically mimicked orbital lymphoma, highlighting the importance of tissue diagnosis in patients presenting with indeterminate orbital masses.

## Case presentation

A 50-year-old man presented to the ophthalmology clinic with a four-year history of progressively worsening right-sided proptosis. The symptom had developed insidiously and gradually increased in prominence over time. There was no documented history of acute visual loss, ocular trauma, constitutional symptoms, or previous orbital surgery. The patient sought medical attention due to the persistent nature of the orbital swelling and the associated cosmetic deformity. His past medical, surgical, family, and medication histories were not contributory. No history suggestive of systemic autoimmune disease, hematological malignancy, or chronic inflammatory disorder was reported at the time of presentation.

Clinical examination demonstrated right eye proptosis with outward displacement of the globe. The remainder of the ophthalmologic examination did not reveal any gross external signs of acute inflammation. No cutaneous lesions or palpable regional lymphadenopathy were noted. General physical examination was otherwise unremarkable, with no evidence of systemic organ involvement.

As part of the diagnostic work-up, computed tomography (CT) of the orbit was performed. Non-contrast CT images demonstrated a hyperdense right extraconal orbital mass predominantly located within the lateral aspect of the orbit. Following intravenous contrast administration, the lesion showed avid enhancement (Figure 1). The mass exhibited indistinct interfaces with adjacent orbital structures, including the enlarged right lacrimal gland as well as the lateral and superior rectus muscles, raising concern for infiltrative pathology. Associated remodeling of the adjacent orbital bone was observed; however, there was no evidence of hyperostosis or frank osseous destruction. The imaging findings suggested a slowly progressive process with local mass effect.

**Figure 1:**
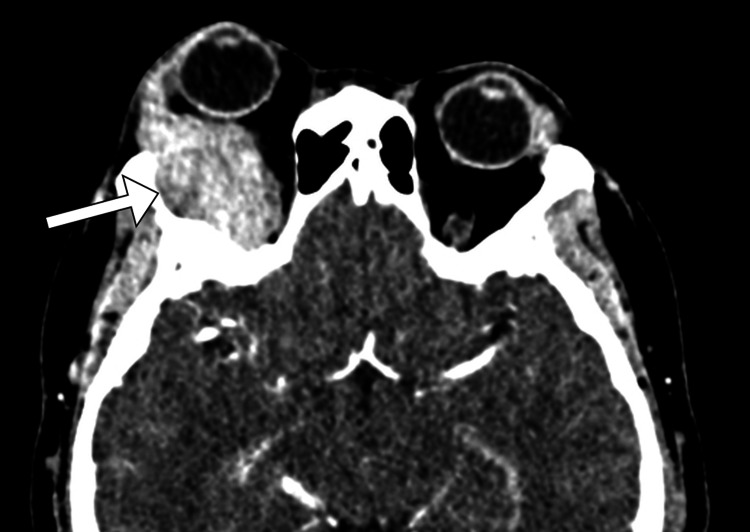
Contrast-enhanced computed tomography of the orbit (axial view) demonstrating a right extraconal orbital mass A hyperdense enhancing right extraconal orbital mass is seen predominantly located along the lateral aspect of the orbit (arrow). The lesion shows indistinct margins with the adjacent lacrimal gland and neighboring extraocular muscles, resulting in right-sided proptosis. Mild adjacent orbital bone remodeling is present without evidence of hyperostosis or osseous destruction.

Subsequent magnetic resonance imaging (MRI) of the orbit was obtained for further tissue characterization and assessment of disease extent. MRI demonstrated that the previously identified right extraconal orbital mass was isointense to skeletal muscle on both T1-weighted and T2-weighted sequences. Diffusion-weighted imaging revealed restricted diffusion within the lesion, and post-contrast sequences demonstrated heterogeneous enhancement. In addition to the dominant lateral orbital lesion, a second smaller extraconal lesion with similar signal characteristics was identified involving the right medial rectus muscle. No focal enhancing intracranial or brain parenchymal abnormalities were detected. The MRI findings reinforced the possibility of a lymphoproliferative or fibroinflammatory orbital process (Figure 2).

**Figure 2:**
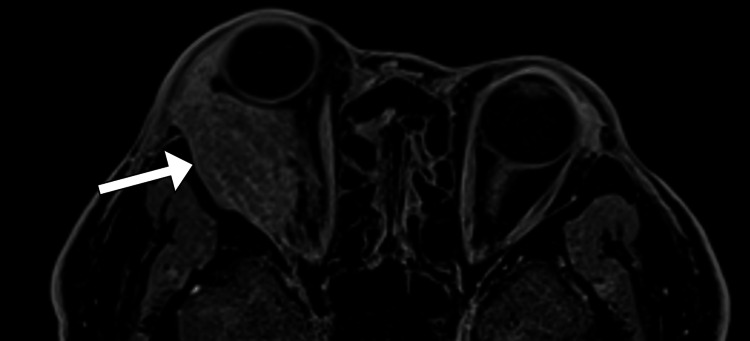
Contrast-enhanced T1-weighted magnetic resonance imaging (axial view) of the orbit demonstrating multifocal orbital involvement Heterogeneous enhancement of the right extraconal orbital mass (arrow) is seen. The lesion is centered within the lateral orbit and extends to involve adjacent orbital structures.

Based on the clinical and radiological findings, the principal differential diagnoses included orbital lymphoma, idiopathic orbital inflammatory disease (orbital pseudotumor), and IgG4-ROD. Orbital lymphoma was strongly considered because of the lesion’s homogeneous soft-tissue appearance, restricted diffusion, and infiltrative pattern involving multiple orbital structures. Idiopathic orbital inflammatory disease was also considered given the involvement of extraocular muscles and lacrimal tissue. IgG4-ROD remained an important diagnostic consideration due to the lacrimal gland enlargement and multifocal orbital involvement.

Given the indeterminate imaging findings and the need for definitive diagnosis, the patient underwent a right anterior orbitotomy with incisional biopsy of the orbital mass. Intraoperatively, the lesion was described as a whitish, firm, and non-capsulated mass. Tissue specimens were submitted for comprehensive histopathological and immunohistochemical evaluation.

Gross pathological examination revealed a brownish nodular tissue specimen with a homogeneous gray-white cut surface. Microscopic examination demonstrated lymphoid tissue adjacent to lacrimal gland lobules, intersected by dense, patternless bands of collagenous fibrosis. Obliterative phlebitis was not identified. The lymphoid component showed mild interfollicular expansion composed of a heterogeneous population of lymphocytes. A prominent lymphoplasmacytic infiltrate was present and consisted of CD3-positive T lymphocytes, CD20-positive B lymphocytes, and CD138-positive mature plasma cells. Germinal centers contained CD10-positive and BCL6-positive B cells with preservation of the follicular dendritic cell meshwork as demonstrated by CD23 immunostaining. BCL2 expression was absent within germinal centers, and the mantle zones remained intact. Ki-67 immunolabeling demonstrated polarized proliferative activity confined to the germinal centers. No atypical lymphoid proliferation, plasma cell dyscrasia, or other evidence of malignancy was identified.

Further immunohistochemical assessment revealed numerous IgG4-positive plasma cells. Areas of highest density demonstrated up to 150 IgG4-positive plasma cells per high-power field, with an IgG4/IgG plasma cell ratio ranging between 50% and 60%. These findings fulfilled accepted histopathological criteria for IgG4-RD. The overall pathological interpretation was highly suggestive of IgG4-ROD involving the lacrimal gland and adjacent orbital soft tissues.

Following multidisciplinary review of the clinical, radiological, and pathological findings, a final diagnosis of IgG4-ROD was established. The diagnosis explained the chronic clinical course, multifocal orbital involvement, lacrimal gland enlargement, and characteristic histopathological features. Importantly, tissue diagnosis excluded orbital lymphoma and other malignant orbital neoplasms that had initially been considered based on imaging appearances.

The postoperative course was uneventful, and no immediate surgical complications were reported. The patient was subsequently referred for continued ophthalmologic and medical evaluation to assess for potential systemic manifestations of IgG4-RD and to guide further therapeutic management. Follow-up assessments demonstrated pathological confirmation of a benign fibroinflammatory IgG4-related process without evidence of malignancy. The case highlights the diagnostic challenge posed by IgG4-ROD, particularly when imaging findings closely mimic orbital lymphoma, emphasizing the critical role of tissue biopsy and immunohistochemical analysis in establishing the correct diagnosis and directing appropriate management.

## Discussion

IgG4-ROD represents a localized manifestation of IgG4-RD, a systemic fibroinflammatory disorder characterized by tumefactive lesions, lymphoplasmacytic infiltration rich in IgG4-positive plasma cells, and varying degrees of fibrosis [1,2]. Since its recognition as a distinct clinicopathological entity, IgG4-RD has emerged as an important diagnostic consideration in patients presenting with orbital masses, particularly those involving the lacrimal glands, extraocular muscles, trigeminal nerve branches, and orbital soft tissues [3,5]. Despite increasing awareness, diagnosis remains challenging because the clinical and radiological manifestations frequently overlap with those of neoplastic, lymphoproliferative, and inflammatory disorders [6-9]. The present case illustrates this diagnostic dilemma, as both CT and MRI findings strongly suggested orbital lymphoma, yet histopathological examination ultimately established the diagnosis of IgG4-ROD.

Orbital involvement is among the most commonly reported manifestations of head and neck IgG4-RD. Patients typically present with slowly progressive proptosis, periorbital swelling, diplopia, or palpable orbital masses, often with a prolonged clinical course [1,3,6]. The chronic four-year history of unilateral proptosis observed in our patient is consistent with the indolent nature of IgG4-ROD reported in previous studies. Such a slow progression may help distinguish IgG4-ROD from aggressive orbital malignancies; however, symptom duration alone is insufficient for diagnosis because low-grade lymphomas can exhibit a similarly indolent clinical behavior.

One of the most notable aspects of this case is the radiological resemblance to orbital lymphoma. CT demonstrated a hyperdense, enhancing extraconal mass with involvement of adjacent structures, while MRI revealed a lesion that was isointense to muscle on both T1- and T2-weighted sequences, exhibited restricted diffusion, and showed heterogeneous post-contrast enhancement. These findings are well recognized in orbital lymphoma and often raise a strong suspicion for a lymphoproliferative neoplasm. Restricted diffusion, in particular, is commonly associated with highly cellular lesions such as lymphoma and therefore may lead clinicians toward a malignant diagnosis. However, increasing evidence suggests that IgG4-ROD can exhibit similar imaging characteristics owing to its dense lymphoplasmacytic infiltrate and fibrotic composition [5,8,10].

The involvement of multiple orbital structures in this patient provides an additional clue favoring IgG4-ROD. The lesion demonstrated an intimate relationship with the lacrimal gland and adjacent extraocular muscles, and a second lesion was identified involving the medial rectus muscle. Lacrimal gland enlargement and extraocular muscle involvement are among the hallmark imaging features of IgG4-ROD and have been reported more frequently than in orbital lymphoma. Nevertheless, substantial overlap exists between these entities, and no single imaging characteristic reliably differentiates them [3,5,7].

The differential diagnosis in this case included orbital lymphoma, idiopathic orbital inflammatory disease (IOID), and IgG4-ROD. Orbital lymphoma remains the most important diagnostic consideration because of its prevalence among adult orbital tumors and its characteristic imaging features [2-8]. Histologically, lymphoma is characterized by a monoclonal lymphoid proliferation with cytologic atypia and architectural effacement, features absent in the present case. Idiopathic orbital inflammatory disease may also mimic IgG4-ROD clinically and radiologically, particularly when extraocular muscles are involved. However, growing evidence suggests that a subset of cases previously classified as idiopathic orbital inflammation may actually represent unrecognized IgG4-RD. The distinction is clinically relevant because the underlying pathogenesis, systemic associations, prognosis, and long-term management strategies differ substantially [6-10].

Histopathological examination remains the cornerstone of diagnosis and was decisive in our patient. The biopsy demonstrated a dense lymphoplasmacytic infiltrate associated with collagenous fibrosis and a markedly increased number of IgG4-positive plasma cells, reaching up to 150 cells per high-power field, with an IgG4+/IgG+ plasma cell ratio of 50-60%. These findings fulfill widely accepted histopathological criteria for IgG4-RD [6-8]. Although obliterative phlebitis is considered one of the classic pathological features of IgG4-RD, its absence does not exclude the diagnosis, particularly in orbital lesions where this finding is reported less frequently than in pancreatic or retroperitoneal disease. Importantly, the preservation of normal follicular architecture, absence of atypical lymphoid populations, and lack of evidence for malignancy effectively excluded orbital lymphoma.

The pathological findings observed in this case also provide insight into the biological behavior of IgG4-RD. The disease is believed to arise from a dysregulated immune response involving B cells, plasmablasts, T-helper 2 cells, regulatory T cells, and profibrotic cytokines [1-5]. This immune-mediated process leads to chronic inflammation and progressive fibrosis, resulting in the formation of tumor-like masses that often mimic malignancy. The dense fibrosis identified within the biopsy specimen likely contributed to the lesion's relatively low T2 signal intensity and firm intraoperative appearance. This radiologic-pathologic correlation highlights the importance of integrating imaging and histological findings when evaluating atypical orbital lesions [2,10].

An important clinical implication of this case is the potential consequence of misdiagnosis. If interpreted solely on the basis of imaging findings, the lesion could have been mistaken for orbital lymphoma, potentially leading to unnecessary oncologic treatment. Conversely, failure to recognize IgG4-ROD may delay appropriate immunosuppressive therapy and permit progression to irreversible fibrosis and organ dysfunction. Therefore, clinicians should maintain a high index of suspicion for IgG4-ROD in patients presenting with chronic orbital masses involving the lacrimal gland and extraocular muscles, particularly when imaging findings are inconclusive.

Another significant consideration is the systemic nature of IgG4-RD. Although our patient presented with isolated orbital disease and no intracranial involvement was identified on MRI, orbital manifestations may represent the initial presentation of a multisystem disorder. Previous studies have demonstrated associations with pancreatic, salivary gland, renal, pulmonary, and lymph node involvement [6-9]. Consequently, diagnosis of IgG4-ROD should prompt a comprehensive evaluation for systemic disease and longitudinal follow-up. Early recognition of extracranial manifestations may facilitate timely intervention and reduce long-term morbidity.

This case underscores the important role of family physicians in the early recognition and longitudinal management of uncommon systemic diseases. Patients with chronic unilateral proptosis or orbital swelling may initially present in primary care, where symptoms can easily be attributed to more common benign conditions. The prolonged clinical course in our patient highlights the need for careful assessment of persistent or progressive symptoms and timely referral for specialist evaluation when red-flag features are present. Furthermore, because IgG4-RD is a multisystem disorder, primary care physicians remain central to ongoing surveillance, coordination of multidisciplinary care, monitoring treatment response, and identifying manifestations in other organ systems that may emerge over time.

## Conclusions

IgG4-ROD should be considered in the differential diagnosis of chronic unilateral proptosis and orbital masses, particularly when symptoms progress slowly over time. For family physicians, persistent orbital symptoms, unexplained glandular enlargement, or atypical inflammatory presentations warrant further investigation and specialist referral. This case highlights the importance of maintaining a broad differential diagnosis, recognizing potential manifestations of systemic disease, and facilitating multidisciplinary care. Histopathological confirmation remains essential for diagnosis, while ongoing primary care follow-up plays a key role in monitoring for systemic involvement and coordinating long-term management.
